# (5-Bromo-2-chloro­phen­yl)(4-ethoxy­phen­yl)methanone

**DOI:** 10.1107/S1600536809047151

**Published:** 2009-11-14

**Authors:** Hua Shao, Guilong Zhao, Wei Liu, Yuli Wang, Weiren Xu

**Affiliations:** a Tianjin Key Laboratory of Molecular Design and Drug Discovery, Tianjin Institute of Pharmaceutical Research, Tianjin 300193, People’s Republic of China

## Abstract

In the title mol­ecule, C_15_H_12_BrClO_2_, the two benzene rings form a dihedral angle of 69.30 (3)°. In the crystal structure, weak inter­molecular C—H⋯O hydrogen bonds link mol­ecules into chains propagating along the *b* axis.

## Related literature

The title compound is an inter­mediate in the synthesis of Dapagliflozin, which exhibits strong biological activity, see Meng *et al.* (2008 ▶). For reference structural data, see Allen *et al.* (1987 ▶).
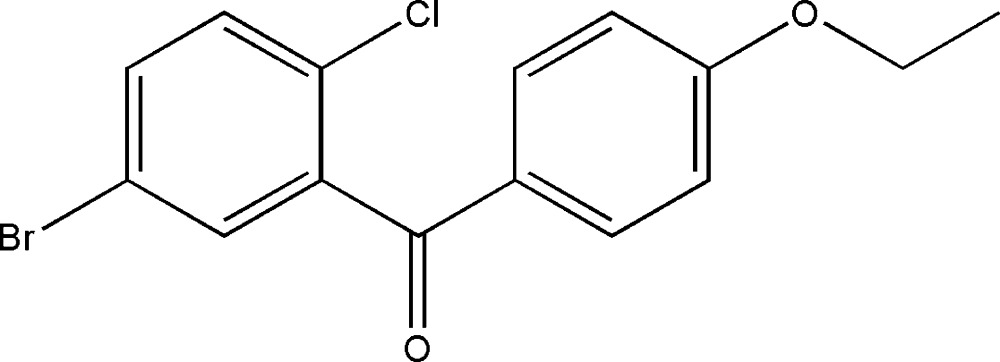


## Experimental

### 

#### Crystal data

C_15_H_12_BrClO_2_
*M* *_r_* = 339.61
Orthorhombic, 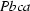

*a* = 9.5979 (19) Å
*b* = 12.951 (3) Å
*c* = 22.457 (5) Å
*V* = 2791.3 (10) Å^3^
*Z* = 8
Mo *K*α radiation
μ = 3.13 mm^−1^
*T* = 153 K
0.22 × 0.18 × 0.12 mm

#### Data collection

Bruker P4 diffractometer
Absorption correction: gaussian (*XSCANS*; Bruker, 1999 ▶) *T*_min_ = 0.546, *T*_max_ = 0.705
17640 measured reflections
2460 independent reflections
2127 reflections with *I* > 2σ(*I*)
*R*_int_ = 0.043

#### Refinement

*R*[*F* ^2^ > 2σ(*F* ^2^)] = 0.032
*wR*(*F* ^2^) = 0.083
*S* = 1.09
2460 reflections
174 parameters
H-atom parameters constrained
Δρ_max_ = 0.56 e Å^−3^
Δρ_min_ = −0.63 e Å^−3^

### 

Data collection: *XSCANS* (Bruker, 1999 ▶); cell refinement: *XSCANS*; data reduction: *XSCANS*; program(s) used to solve structure: *SHELXS97* (Sheldrick, 2008 ▶); program(s) used to refine structure: *SHELXL97* (Sheldrick, 2008 ▶); molecular graphics: *SHELXTL* (Sheldrick, 2008 ▶); software used to prepare material for publication: *SHELXTL*.

## Supplementary Material

Crystal structure: contains datablocks I, global. DOI: 10.1107/S1600536809047151/cv2652sup1.cif

Structure factors: contains datablocks I. DOI: 10.1107/S1600536809047151/cv2652Isup2.hkl

Additional supplementary materials:  crystallographic information; 3D view; checkCIF report

## Figures and Tables

**Table 1:** Hydrogen-bond geometry (Å, °)

*D*—H⋯*A*	*D*—H	H⋯*A*	*D*⋯*A*	*D*—H⋯*A*
C10—H10⋯O1^i^	0.93	2.50	3.369 (3)	156

Symmetry code: (i) 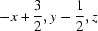.

**Fig. 1.**
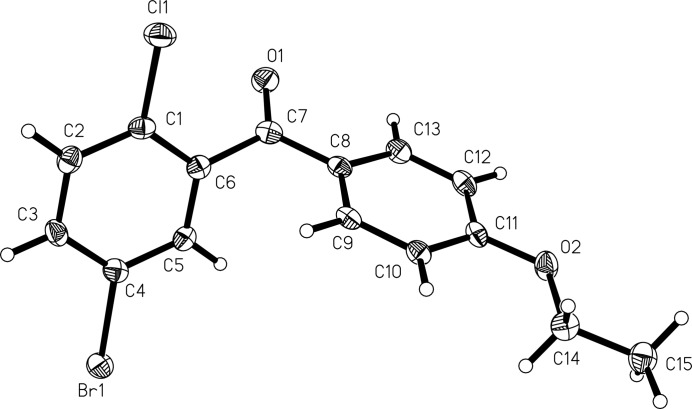
View of the title compound, with displacement ellipsoids drawn at the 40% probability level.

## supplementary crystallographic information

### Comment

Dapagliflozin is an anti-diabetic agent through the inhibition of renal SGLT2, which is developed by Bristol-Myers Squibb Company, and now it is in the phase III clinical trial (Meng *et al.*, 2008). During the development of our own SGLT2 inhibitors as anti-diabetic agents, Dapagliflozin was synthesized as the positive control in the bioactivity screening, and the title compound, (I), was prepared as an intermediate. The crystallographic analysis of (I) confirms the molecular structures of the title compound and Dapagliflozin.

In (I) (Fig. 1), all bond lengths are normal and in a good agreement with those reported previously (Allen *et al.*, 1987). Two benzene rings (C1—C6 and C8—C13) form a dihedral angle of 69.30 (3) °. In the crystal structure, weak intermolecular C—H···O hydrogen bonds (Table 1) link molecules into chains along axis *b*.

### Experimental

A round-bottomed flask was charged with 2.36 g (10 mmol) of 5-bromo-2-chlorobenoic acid, 1 drop of DMF, 1.27 g (10 mmol) of oxalyl chloride and 3 ml of dried dichloromethane, and the mixture was stirred at room temperature over night until a clear solution formed. The reaction mixture was evaporated on a rotary evaporator to give crude 5-bromo-2-chlorobenzoic acid, which was dissolved in 15 ml of dried dichloromethane. The solution thus obtained was stirred while being cooled with an ice-salt mixture, and 1.22 g (10 mmol) of phenetole was added followed by addition of 1.60 g (12 mmol) of anhydrous aluminium chloride in a portionwise manner. The resulting mixture was stirred at this temperature for 1 h and poured into 150 ml of ice-water. The mixture thus formed was exacted with three 50-ml portions of dichloromethane, and the combined exacts were washed with saturated brine, dried over sodium sulfate and evaporated on a rotary evaporator to afford the crude title compound. Pure title compound was obtained by column chromatography (2.86 g 84.2%). Crystals suitable for X-ray diffraction were obtained through slow evaporation of a solution of the pure title compound in dichloromethane/ethyl acetate/petroleum ether (2/1/3 by volume).

### Refinement

All C-bound H atoms were placed in calculated positions, with C—H = 0.93–0.97 Å, and included in the final cycles of refinement using a riding model, with *U*_iso_(H) = 1.2*U*_eq_(C) for the aryl and methylene H atoms and 1.5*U*_eq_(C) for the methyl H atoms.

### Crystal data

**Table:**

C_15_H_12_BrClO_2_	*F*(000) = 1360
*M**_r_* = 339.61	*D*_x_ = 1.616 Mg m^−^^3^
Orthorhombic, *P**b**c**a*	Mo *K*α radiation, λ = 0.71073 Å
Hall symbol: -P 2ac 2ab	Cell parameters from 7090 reflections
*a* = 9.5979 (19) Å	θ = 2.6–27.9°
*b* = 12.951 (3) Å	µ = 3.13 mm^−^^1^
*c* = 22.457 (5) Å	*T* = 153 K
*V* = 2791.3 (10) Å^3^	Block, colourless
*Z* = 8	0.22 × 0.18 × 0.12 mm

### Data collection

**Table:**

Bruker P4 diffractometer	2460 independent reflections
Radiation source: sealed tube	2127 reflections with *I* > 2σ(*I*)
graphite	*R*_int_ = 0.043
ω scans	θ_max_ = 25.0°, θ_min_ = 2.8°
Absorption correction: gaussian (*XSCANS*; Bruker, 1999)	*h* = −11→10
*T*_min_ = 0.546, *T*_max_ = 0.705	*k* = −15→12
17640 measured reflections	*l* = −26→23

### Refinement

**Table:**

Refinement on *F*^2^	Secondary atom site location: difference Fourier map
Least-squares matrix: full	Hydrogen site location: inferred from neighbouring sites
*R*[*F*^2^ > 2σ(*F*^2^)] = 0.032	H-atom parameters constrained
*wR*(*F*^2^) = 0.083	*w* = 1/[σ^2^(*F*_o_^2^) + (0.0488*P*)^2^ + 0.2837*P*] where *P* = (*F*_o_^2^ + 2*F*_c_^2^)/3
*S* = 1.09	(Δ/σ)_max_ = 0.001
2460 reflections	Δρ_max_ = 0.56 e Å^−^^3^
174 parameters	Δρ_min_ = −0.62 e Å^−^^3^
0 restraints	Extinction correction: *SHELXL97* (Sheldrick, 2008), Fc^*^=kFc[1+0.001xFc^2^λ^3^/sin(2θ)]^-1/4^
Primary atom site location: structure-invariant direct methods	Extinction coefficient: 0.0024 (4)

### Special details

**Table:**

Geometry. All e.s.d.'s (except the e.s.d. in the dihedral angle between two l.s. planes) are estimated using the full covariance matrix. The cell e.s.d.'s are taken into account individually in the estimation of e.s.d.'s in distances, angles and torsion angles; correlations between e.s.d.'s in cell parameters are only used when they are defined by crystal symmetry. An approximate (isotropic) treatment of cell e.s.d.'s is used for estimating e.s.d.'s involving l.s. planes.
Refinement. Refinement of *F*^2^ against ALL reflections. The weighted *R*-factor *wR* and goodness of fit *S* are based on *F*^2^, conventional *R*-factors *R* are based on *F*, with *F* set to zero for negative *F*^2^. The threshold expression of *F*^2^ > σ(*F*^2^) is used only for calculating *R*-factors(gt) *etc*. and is not relevant to the choice of reflections for refinement. *R*-factors based on *F*^2^ are statistically about twice as large as those based on *F*, and *R*- factors based on ALL data will be even larger.

### Fractional atomic coordinates and isotropic or equivalent isotropic displacement parameters (Å^2^)

**Table:**

	*x*	*y*	*z*	*U*_iso_*/*U*_eq_	
Br1	1.01219 (3)	0.15934 (2)	0.193829 (13)	0.03274 (14)	
Cl1	0.65219 (8)	0.50193 (5)	0.32750 (3)	0.0359 (2)	
O1	0.9357 (2)	0.46850 (12)	0.39989 (8)	0.0332 (5)	
O2	0.8726 (2)	0.06639 (12)	0.55900 (7)	0.0302 (4)	
C1	0.7603 (3)	0.41473 (16)	0.29098 (12)	0.0241 (6)	
C2	0.7414 (3)	0.39945 (17)	0.23067 (12)	0.0270 (6)	
H2	0.6765	0.4389	0.2100	0.032*	
C3	0.8190 (3)	0.32545 (18)	0.20097 (11)	0.0265 (6)	
H3	0.8088	0.3162	0.1601	0.032*	
C4	0.9117 (3)	0.26556 (17)	0.23294 (10)	0.0252 (6)	
C5	0.9313 (3)	0.28109 (17)	0.29345 (10)	0.0243 (6)	
H5	0.9940	0.2399	0.3143	0.029*	
C6	0.8577 (3)	0.35790 (17)	0.32320 (11)	0.0234 (6)	
C7	0.8941 (3)	0.38177 (18)	0.38710 (11)	0.0253 (6)	
C8	0.8846 (3)	0.29806 (17)	0.43151 (10)	0.0227 (6)	
C9	0.8050 (3)	0.20985 (17)	0.42111 (11)	0.0226 (6)	
H9	0.7571	0.2037	0.3853	0.027*	
C10	0.7954 (3)	0.13140 (17)	0.46257 (11)	0.0232 (6)	
H10	0.7399	0.0739	0.4552	0.028*	
C11	0.8703 (3)	0.13978 (17)	0.51571 (10)	0.0240 (6)	
C12	0.9505 (3)	0.22788 (19)	0.52704 (11)	0.0285 (6)	
H12	1.0000	0.2335	0.5625	0.034*	
C13	0.9559 (3)	0.30605 (19)	0.48567 (11)	0.0273 (6)	
H13	1.0077	0.3651	0.4938	0.033*	
C14	0.7954 (3)	−0.02766 (17)	0.54863 (12)	0.0298 (6)	
H14A	0.8222	−0.0581	0.5109	0.036*	
H14B	0.6962	−0.0135	0.5477	0.036*	
C15	0.8293 (3)	−0.1000 (2)	0.59905 (12)	0.0348 (7)	
H15A	0.9277	−0.1135	0.5994	0.052*	
H15B	0.7796	−0.1636	0.5938	0.052*	
H15C	0.8025	−0.0689	0.6361	0.052*	

### Atomic displacement parameters (Å^2^)

**Table:**

	*U*^11^	*U*^22^	*U*^33^	*U*^12^	*U*^13^	*U*^23^
Br1	0.0368 (2)	0.0330 (2)	0.0284 (2)	0.00518 (11)	0.01330 (12)	−0.00036 (11)
Cl1	0.0359 (5)	0.0346 (4)	0.0371 (4)	0.0117 (3)	−0.0049 (3)	−0.0100 (3)
O1	0.0377 (13)	0.0273 (9)	0.0347 (11)	−0.0038 (8)	−0.0067 (9)	−0.0030 (8)
O2	0.0342 (12)	0.0340 (9)	0.0223 (9)	−0.0072 (8)	−0.0035 (8)	0.0017 (8)
C1	0.0223 (15)	0.0195 (12)	0.0306 (14)	−0.0020 (10)	0.0034 (11)	−0.0013 (10)
C2	0.0260 (16)	0.0262 (13)	0.0290 (14)	−0.0026 (11)	−0.0032 (11)	0.0038 (11)
C3	0.0326 (17)	0.0286 (13)	0.0183 (13)	−0.0058 (11)	0.0038 (11)	−0.0002 (10)
C4	0.0243 (16)	0.0258 (12)	0.0256 (13)	−0.0031 (11)	0.0086 (12)	−0.0005 (10)
C5	0.0210 (16)	0.0251 (13)	0.0269 (13)	0.0006 (11)	0.0015 (11)	0.0045 (10)
C6	0.0212 (15)	0.0227 (12)	0.0263 (13)	−0.0030 (10)	0.0020 (11)	0.0016 (10)
C7	0.0180 (15)	0.0274 (13)	0.0304 (14)	0.0000 (11)	0.0007 (12)	−0.0044 (11)
C8	0.0186 (15)	0.0261 (12)	0.0234 (13)	0.0031 (10)	0.0011 (11)	−0.0052 (10)
C9	0.0189 (15)	0.0277 (13)	0.0212 (13)	0.0021 (10)	−0.0011 (10)	−0.0073 (10)
C10	0.0209 (15)	0.0263 (12)	0.0224 (13)	−0.0034 (10)	0.0005 (11)	−0.0044 (10)
C11	0.0214 (16)	0.0327 (13)	0.0181 (13)	0.0016 (11)	0.0024 (11)	−0.0019 (11)
C12	0.0279 (16)	0.0370 (15)	0.0206 (13)	−0.0014 (12)	−0.0035 (12)	−0.0070 (11)
C13	0.0247 (16)	0.0301 (13)	0.0271 (14)	−0.0033 (11)	−0.0008 (12)	−0.0049 (11)
C14	0.0249 (17)	0.0320 (13)	0.0324 (15)	−0.0038 (11)	−0.0002 (12)	0.0001 (11)
C15	0.0309 (18)	0.0382 (15)	0.0353 (16)	−0.0014 (12)	0.0028 (13)	0.0028 (12)

### Geometric parameters (Å, °)

**Table:**

Br1—C4	1.896 (2)	C8—C9	1.394 (3)
Cl1—C1	1.739 (2)	C8—C13	1.399 (3)
O1—C7	1.226 (3)	C9—C10	1.381 (3)
O2—C11	1.360 (3)	C9—H9	0.9300
O2—C14	1.444 (3)	C10—C11	1.397 (3)
C1—C2	1.381 (4)	C10—H10	0.9300
C1—C6	1.392 (4)	C11—C12	1.400 (3)
C2—C3	1.385 (4)	C12—C13	1.375 (4)
C2—H2	0.9300	C12—H12	0.9300
C3—C4	1.382 (4)	C13—H13	0.9300
C3—H3	0.9300	C14—C15	1.505 (3)
C4—C5	1.386 (3)	C14—H14A	0.9700
C5—C6	1.391 (3)	C14—H14B	0.9700
C5—H5	0.9300	C15—H15A	0.9600
C6—C7	1.509 (3)	C15—H15B	0.9600
C7—C8	1.476 (3)	C15—H15C	0.9600
			
C11—O2—C14	117.77 (19)	C10—C9—H9	119.1
C2—C1—C6	121.5 (2)	C8—C9—H9	119.1
C2—C1—Cl1	118.49 (19)	C9—C10—C11	119.0 (2)
C6—C1—Cl1	119.9 (2)	C9—C10—H10	120.5
C1—C2—C3	120.1 (2)	C11—C10—H10	120.5
C1—C2—H2	120.0	O2—C11—C10	124.4 (2)
C3—C2—H2	120.0	O2—C11—C12	115.5 (2)
C4—C3—C2	119.0 (2)	C10—C11—C12	120.1 (2)
C4—C3—H3	120.5	C13—C12—C11	119.9 (2)
C2—C3—H3	120.5	C13—C12—H12	120.1
C3—C4—C5	121.0 (2)	C11—C12—H12	120.1
C3—C4—Br1	119.63 (18)	C12—C13—C8	121.0 (2)
C5—C4—Br1	119.37 (19)	C12—C13—H13	119.5
C4—C5—C6	120.4 (2)	C8—C13—H13	119.5
C4—C5—H5	119.8	O2—C14—C15	107.0 (2)
C6—C5—H5	119.8	O2—C14—H14A	110.3
C5—C6—C1	118.0 (2)	C15—C14—H14A	110.3
C5—C6—C7	119.1 (2)	O2—C14—H14B	110.3
C1—C6—C7	122.8 (2)	C15—C14—H14B	110.3
O1—C7—C8	122.3 (2)	H14A—C14—H14B	108.6
O1—C7—C6	119.1 (2)	C14—C15—H15A	109.5
C8—C7—C6	118.6 (2)	C14—C15—H15B	109.5
C9—C8—C13	118.3 (2)	H15A—C15—H15B	109.5
C9—C8—C7	121.5 (2)	C14—C15—H15C	109.5
C13—C8—C7	120.2 (2)	H15A—C15—H15C	109.5
C10—C9—C8	121.8 (2)	H15B—C15—H15C	109.5
			
C6—C1—C2—C3	0.7 (4)	O1—C7—C8—C9	163.0 (2)
Cl1—C1—C2—C3	−175.51 (19)	C6—C7—C8—C9	−20.0 (4)
C1—C2—C3—C4	2.0 (4)	O1—C7—C8—C13	−16.7 (4)
C2—C3—C4—C5	−2.3 (4)	C6—C7—C8—C13	160.3 (2)
C2—C3—C4—Br1	176.92 (19)	C13—C8—C9—C10	0.0 (4)
C3—C4—C5—C6	−0.2 (4)	C7—C8—C9—C10	−179.8 (2)
Br1—C4—C5—C6	−179.38 (19)	C8—C9—C10—C11	−1.6 (4)
C4—C5—C6—C1	2.9 (4)	C14—O2—C11—C10	1.7 (4)
C4—C5—C6—C7	−172.4 (2)	C14—O2—C11—C12	−177.9 (2)
C2—C1—C6—C5	−3.2 (4)	C9—C10—C11—O2	−177.9 (2)
Cl1—C1—C6—C5	173.05 (19)	C9—C10—C11—C12	1.7 (4)
C2—C1—C6—C7	171.9 (2)	O2—C11—C12—C13	179.4 (2)
Cl1—C1—C6—C7	−11.8 (3)	C10—C11—C12—C13	−0.2 (4)
C5—C6—C7—O1	118.6 (3)	C11—C12—C13—C8	−1.4 (4)
C1—C6—C7—O1	−56.4 (4)	C9—C8—C13—C12	1.6 (4)
C5—C6—C7—C8	−58.5 (3)	C7—C8—C13—C12	−178.7 (2)
C1—C6—C7—C8	126.5 (3)	C11—O2—C14—C15	173.1 (2)

### Hydrogen-bond geometry (Å, °)

**Table:**

*D*—H···*A*	*D*—H	H···*A*	*D*···*A*	*D*—H···*A*
C10—H10···O1^i^	0.93	2.50	3.369 (3)	156

Symmetry codes: (i) −*x*+3/2, *y*−1/2, *z*.
